# Determining why continuous cropping reduces the production of the morel *Morchella sextelata*

**DOI:** 10.3389/fmicb.2022.903983

**Published:** 2022-09-12

**Authors:** Liu Wei-Ye, Guo Hong-Bo, Bi Ke-Xin, Sibirina Lidiya Alekseevna, Qi Xiao-Jian, Yu Xiao-Dan

**Affiliations:** ^1^College of Biological Science and Technology, Shenyang Agricultural University, Shenyang, China; ^2^College of Life Engineering, Shenyang Institute of Technology, Fushun, China; ^3^Primorye State Agricultural Academy, Ussuriisk, Russia; ^4^Federal Scientific Center of the East Asia Terrestrial Biodiversity, Far Eastern Branch of Russian Academy of Sciences, Vladivostok, Russia

**Keywords:** *Morchella*, continuous cropping, microbial community, pathogenic fungi, function prediction

## Abstract

Artificial cultivation of *Morchella sextelata* and other morels is expanding in China, but continuous cropping reduces *Morchella* for unknown reasons. Here, we investigated soil that had been used or not used for *M. sextelata* cultivation for 0, 1, and 2 years. We found that the continuous cropping of *M. sextelata* substantially reduced the pH and the nutrient content of the hyphosphere soil and increased sclerotia formation by *M. sextelata*. Changes in the structure of bacterial and fungal communities were associated with levels of available nitrogen (N) and phosphorus in the soil. With continuous cropping, the richness and diversity of fungal and bacterial communities increased, but the abundance of *Bacillus* and *Lactobacillus* decreased and the abundance of pathogenic fungi increased. FAPROTAX analysis indicated that N cycle functions were enriched more with than without continuous cultivation, and that enrichment of N cycle and sulfate respiration functions was higher in the second than in the first year of cultivation. FunGuild analysis indicated that the functions related to pathotrophs and wood saprotrophs were enriched by *M. sextelata* cultivation. Overall, the results suggest that continuous cropping may reduce *M. sextelata* production by acidifying the soil and increasing the abundance of pathogenic fungi. Additional research is needed to determine whether increases in the abundance of pathogenic fungi and changes in soil chemistry result in the declines in production that occur with continuous *M. sextelata* cultivation.

## Introduction

In addition to being flavorful, the fruiting bodies of morel mushrooms (*Morchella* spp., *Ascomycetes*) contain a variety of nutrients and antioxidant, antibacterial, and immune-regulating compounds. As a result, *Morchella* spp. are highly valued (Li et al., 2017; Tietel and Masaphy, 2018; Meng et al., 2019; Badshah et al., 2021). The market demand for morels has been rising but cannot be met by wild resources (Du et al., 2016; Yang et al., 2019), such that morel cultivation is necessary. In recent years, the cultivation of *Morchella* spp. in China has gradually increased but so have associated problems including outbreaks of diseases and pests that reduce yields (Guo et al., 2016; Liu et al., 2021b). Guo et al. (2016) reported a serious rot disease of *Morchella* caused by *Fusarium incarnatum-F. equiseti* species complex from China. The natural incidence rate of the disease was more than 30%. *Fusarium nematophilum* was also identified as one of the main pathogens of stipe rot disease, which caused heavy losses in *Morchella sextelata* cultivation (Liu et al., 2021b).

The term “continuous cropping” refers to the practice of cultivating the same crop in the same place for more than 1 year. In addition to increasing the incidence and severity of diseases and pests, continuous cropping can alter soil physicochemical properties and soil microbial communities in ways that are detrimental to the growth of plant crop (Ren et al., 2015; Dong et al., 2017; Shen et al., 2018). Continuous cropping obstacle have been widely studied in plants, primarily based on high-throughput amplicon sequencing and microbiome analyses. The continuous cropping of sugar beet, for example, reduces the abundance of beneficial soil microorganisms, increases the abundance of pathogenic soil microorganisms, and leads to the accumulation of compounds responsible for allelopathic autotoxicity (Huang et al., 2019, 2021). For sugarcane, continuous cropping reduces the abundance of soil bacteria related to the nitrogen (N) and sulfur cycle in hyphosphere soil and increases the abundance of plant-pathogenic bacteria (Pang et al., 2021). In the case of tea, continuous cropping causes changes in the soil fungal community that were correlated with changes in soil pH and exchangeable aluminum content and the enrichment of functions related to pathotroph fungi (Li et al., 2020). Functional pathway analyses have indicated that the problems resulting from the continuous cropping of plants involve changes in soil physicochemical properties, in microbial community structure, and autotoxicity (Coskun et al., 2017; Cheng et al., 2020; Li et al., 2021a).

Regarding the cultivation of edible or medicinal mushrooms, the continuous cropping of *Agaricus bisporus* was found to increase the abundance of *Penicillium* and *Mucor* species in soil (Huang, 2006). Problems resulting from the continuous cropping of *Ganoderma lingzhi* may result from *Penicillium* spp. (Yuan et al., 2019). To our knowledge, problems resulting from the continuous cropping of *Morchella* spp. have not been previously investigated.

Consequently, it was hypothesized that the continuous cropping obstacle of *Morchella* is caused by the inhibition of rhizosphere exudates and the accumulation of pathogenic microorganisms. To test the hypothesis, in the current study, high throughput amplicon sequencing was used to assess fungal (ITS rDNA) and prokaryotic (16S rDNA) communities from the morel cultivation environment. We would determine whether problems in the continuous cropping of the morel *Morchella sextelata* are associated with changes in the composition and functional pathways of the soil microbial community, soil physicochemical properties, and soil allelochemicals.

## Materials and methods

### Description of the site, soil, and collection of samples

**FIGURE 1:**
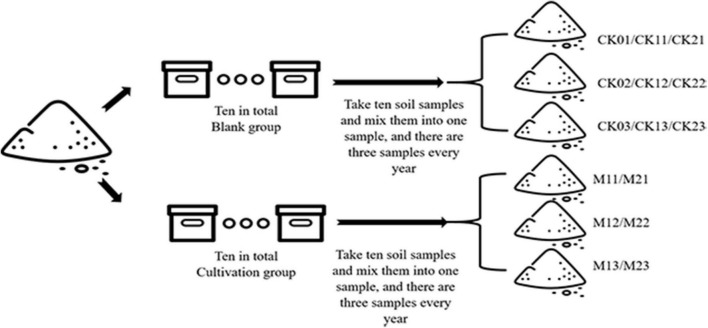
Diagram of the experimental procedure.

The experiment was conducted in 20 wooden boxes (83 cm x 58 cm x 51 cm) that were kept outside in Shenyang City, Liaoning province, China (123°45′N, 41°80′E). The experiment was conducted as described in Figure 1. Soil was collected Tian-zhu Mountain in Shenyang City and was amended with 10% humus. First, the soil was exposed, sieved (2 mm), and then evenly divided into 20 parts, and one part (≈ 245 cm^3^) was placed in each of 20 boxes (83 cm × 58 cm × 51 cm). All the boxes were placed in a greenhouse in experimental base of Shenyang Agriculture University. Among the 20 boxes, 10 boxes were cultivated with *M. sextelata*, and 10 boxes without *M. sextelata* were used as controls. The cultivation method followed the previous studies (Liu, 2017; Benucci et al., 2019). The criteria for the determination of *Morchella* infection is that the fruiting bodies of *Morchella* have lesions, damage, rot, developmental deformities, death, etc., which are regarded as *Morchella* infection (Liu, 2017).

*M. sextelata* was planted in November of 2019 and 2020 and was harvested about 3 months later, in February of 2020 and 2021. The process of soil sampling is outlined in Table 1. Soil samples (10 g) were collected from each box and were combined, mixed, and divided into three samples for CK boxes (without *M. sextelata*) and three samples for M boxes (with *M. sextelata*). For CK boxes, this was done at the start of the experiment (samples CK01, CK02, and CK03) and again in February 2020 and February 2021 (see Table 1). For M boxes, this was done in February 2020 and February 2021 (see Table 1). Each of the nine CK samples and each of the six M samples was divided into three parts: one part was dried for analysis of soil physicochemical properties; a second part was dried to determine the autotoxicity of soil allelochemicals; and a third part was stored at −80°C and was used for extraction of soil DNA.

**TABLE 1:** Soil sample abbreviations and descriptions.

Sample abbreviation	Sample description
CK0	Soil before addition of with *M. sextelata* (November 2019)
CK1	Soil 1 year after not adding *M. sextelata* (February 2020)
CK2	Soil 2 years after not adding *M. sextelata* (February 2021)
M1	Soil 1 year after adding *M. sextelata* (February 2020)
M2	Soil 2 years after adding *M. sextelata* (February 2021)

CK0, CK1, and CK2 are collectively referred to as CKs, and M1 and M2 are collectively referred to as Ms.

### Analysis of soil physicochemical properties and determination of the autotoxicity of soil allelochemicals

All the soil physicochemical properties in this study were testedy in Analytical and Testing Center, Shenyang Agricultural University. The standards for the physicochemical properties were listed as follows. The pH value: NY/T 1121.2-2006; total nitrogen (TN): NY/T 53-1987; total potassium (TK): NY/T 87-1988; total phosphorus (TP): NY/T 88-1988; available nitrogen (AN): NY/T 1121.7-2014; available potassium (AK): NY/T 889-2004; available phosphorus (AP): NY/T 1121.7-2014; organic matter (OM): NY/T 1121.6-2006; humic substances (HS): NY/T 1867-2010. The steps for testing the autotoxicity of soil allelochemicals were as follow: A 0.5-g subsample of each replicate of the five samples (CK0, CK1, CK2, M1, and M2) was sterilized at 121°C for 20 min. After the five sterilized subsamples were placed in five equally spaced locations along the edge of a 9-cm-diameter Petri plate (one subsample per sample per location) containing potato dextrose agar (PDA), the center of each place was inoculated with *M. sextelata*. The plates (10 per sample type) were kept at 25 °C, and the radial growth of *M. sextelata* toward each subsample was measured after 4, 6, 8 days, respectively.

### DNA extraction, PCR amplification, and Illumina MiSeq sequencing

The procedures described in this section were performed by Novogene Bioinformatics Technology Co., Ltd. Total genome DNA from samples was extracted using the CTAB method. 16S rRNA genes of distinct regions (16S V3-V4) were amplified with the 16S-specific primers V3-V4:341F (5′-CCTAYGGGRBGCASCAG-3′) and 806R (5′-GGACTACNNGGGTATCTAAT-3′). ITS genes of distinct regions were amplified using the specific primers ITS2: ITS3-2024F (5′-GCATCGATGAAGAACGCAGC-3′) and ITS4-2409R (5′-TCCTCC GCTTATTGATATGC-3′). All PCR reactions were carried out with 15 μL of Phusion^®^ High-Fidelity PCR Master Mix (New England Biolabs), 0.2 μM of forward and reverse primers, and about 10 ng of template DNA. Thermal cycling consisted of initial denaturation at 98°C for 1 min; followed by 30 cycles of denaturation at 98°C for 10 s, annealing at 50°C for 30 s, and elongation at 72°C for 30 s; and a final extension at 72°C for 5 min. PCR products were mixed in equidensity ratios. The mixture of PCR products was then purified with the Qiagen Gel Extraction Kit (Qiagen, Germany). Sequencing libraries were generated using the TruSeq^®^ DNA PCR-Free Sample Preparation Kit (Illumina, United States) following the manufacturer’s recommendations, and index codes were added. Library quality was assessed with the Qubit @ 2.0 Fluorometer (Thermo Fisher Scientific) and Agilent Bioanalyzer 2100 system. Finally, the library was sequenced on an Illumina NovaSeq platform, and 250-bp paired-end reads were generated.

### Operational taxonomic unit cluster and species annotation

The Uparse algorithm (Haas et al., 2011) was used to cluster all effective tags of all samples. By default, the sequences were clustered into operational taxonomic units (OTUs) with 97% identity. At the same time, the representative sequences of OTUs were selected. The sequence with the highest frequency in OTUs was selected as the representative sequence of OTUs. Species annotation of bacterial OTUs sequences was performed using the mothur method and the SSUrRNA database (Wang et al., 2007) of SILVA138 (Edgar, 2013) to obtain taxonomic information (the threshold was set at 0.8-1.0). Species annotation of fungal OTUs sequences was performed using the blast method^1^ (Altschul et al., 1990) in Qiime software (Version 1.9.1) and the Unite (v8.2) database^2^ (Kõljalg et al., 2013). The community composition of each sample was then determined at each taxonomic level: kingdom, phylum, class, order, family, genus, and species. MUSIC software (Quast et al., 2013) was used for fast multi sequence alignment to determine the phylogenetic relationships among all OTU representative sequences. Finally, the data of each sample were homogenized, and the data with the least amount of data in the sample was used as the standard for homogenization. The subsequent Alpha diversity and Beta diversity analyses were based on the homogenized data.

### Alpha diversity

Alpha diversity was applied to analyze the complexity of species diversity for a sample through 6 indices, including Observed-species, Chao1, Shannon, Simpson. All these indices in our samples were calculated with QIIME (Version 1.7.0) and were displayed with R software (Version 2.15.3).

### Beta diversity

Beta diversity analysis was used to evaluate community differences between groups. Beta diversity on both weighted and unweighted unifrac were calculated by QIIME software (Version 1.9.1). Non-metric multi-dimensional scaling (NMDS) and principal component analysis (PCA) diagrams were drawn with R software (Version 2.15.3). The vegan package of R software was used for NMDS analysis, and the ade4 package and ggplot2 package of R software were used for PCA analysis. Unweighted pair-group method with Arithmetic Means (UPGMA) clustering was performed as a type of hierarchical clustering method to interpret the distance matrix using average linkage and was conducted with QIIME software (Version 1.9.1).

### Correlation analysis of environmental factors

For canonical correlation analysis (CCA) and redundancy analysis (RDA), the CCA and RDA functions in the vegan package were used for ranking analysis. The *r*^2^ and *P*-values for the relationships between each environmental factor and species distribution were calculated with the envfit function, and environmental factors with significant *P*-values were then screened for CCA and RDA analysis. After the BioEnv function in vegan package was used to identify the environmental factors or combinations with the greatest Spearman correlation with the species matrix, the identified factors were subjected to targeted CCA and RDA analysis. Variance inflation factor (Vif) used the vif.cca function in vegan package for screening the environmental factors with redundancy constraints, and then non-redundant environmental factors were used for CCA and RDA analysis. According to the results of a degraded response analysis (DCA), RDA analysis was selected to reflect the relationship between soil microorganisms and environmental factors. The corr.test function of the psych package in R was used to calculate the Spearman correlation coefficients between species and environmental factors, and the pheatmap function in pheatmap package was then used for visualization.

### Functional prediction

Phylogenetic investigation of communities by reconstruction of unobserved states (PICRUSt) was used to identify the gene function spectrum of their common ancestor based on the gene information on the tree of OTUs in the Greenenes database; And analyze the gene function spectrum of other unmeasured species in the Greenenes database, constructs the gene function prediction spectrum of the whole lineage of Archaea and bacterial domain; Finally, maps the composition of the sequenced soil microorganisms into the database, we predicted the metabolic function of soil microorganisms. FAPROTAX was the environmental function database of prokaryotes, which classified the ecological role of bacteria and archaea in the environment according to the published literature evidence, and summarized it into FAPROTAX database. Based on the annotation results of amplified sub species, the database can be queried to obtain the environmental function information of species supported by existing literature.

FunGuid was the environmental function database of fungi. Based on the existing literature, the ecological functions of fungi were classified and the FunGuid database was constructed. Based on the species information obtained by amplicon analysis, the ecological functions of existing species in the literature can be forecasted.

### Statistical analysis

Soil physicochemical properties were analyzed using one-way analysis of variance (ANOVA), followed by Duncan’s multiple range tests. The Paired Samples Test showed significant differences in yield and infection rates between M1 and M2. For all parameters, data were calculated using SPSS Statistics software version 17.0 (SPSS Inc., United States). Differences at *P* < 0.05 were regarded as statistically significant.

## Results

### Morchella sextelata fruiting body production

After 2 years of continuous cultivation, we counted the production of 2-year fruiting body and diseased fruiting body (Supplementary Table 1.1). The production of M2 was significantly lower than that of M1 (113.0 g for M2 vs. 221.7 g for M1). However, the infection rate of fruiting bodies of M2 was significantly higher than that of fruiting bodies of M1 (35.9% for M2 vs. 9.0% for M1). The significant differences in yield and infection rates showed in Supplementary Tables 1.2, 1.3.

### Soil physicochemical properties and the autotoxicity of allelochemicals

Soil pH and the contents of AK, AP, and TK decreased significantly or tended to decrease over time (Table 2). AN, TN, and OM did not significantly differ between CK0 and M1, but were significantly lower in M2 than in CK0. Four days after Petri plates with the five kinds of soil samples were inoculated with *M. sextelata* (Supplementary Figure 1), the mycelium of the fungus had grown over the entire surface of all plates. At the stage of sclerotia formation, however, sclerotia formation was particularly vigorous toward the M2 soil sample.

**TABLE 2:** Effects of continuous cropping of *Morchella sextelata* on the physicochemical properties of hyphosphere soil.

Sample type	AK (mg/kg)	AP (mg/kg)	AN (mg/kg)	TK (%)	TP (%)	TN (%)	OM (g/kg)	pH	HS (%)
CK0	122.9a	88.7a	131.6a	1.79a	0.090a,b	0.157	33.0a,b	8.18a	1.61a,b
M1	101.3c	63.9c	131.3a	1.67c	0.071b	0.152	31.8b	7.75b	1.71a
M2	90.3d	55.5d	120.3b	1.59d	0.087a,b	0.147	29.0c	7.52c	1.47c
CK1	120.3b	86.5b	132.5a	1.77b	0.092a	0.156	34a	8.12a	1.56b,c
CK2	121.2a,b	88.5a	132.7a	1.76b	0.100a	0.158	34.2a	8.10a	1.54b,c

Values are means of three replicates. Means in a column followed by different letters are significantly different at P ≤ 0.05 according to the Duncan’s multiple range test.

### Illumina sequencing data analysis

By splicing reads, we measured on average of 106,033 tags for each bacterial sample; 90,433 raw tags were obtained through quality control, with 65,307 effective tags and a 61.65% effective rate of quality control. An average of 102,621 tags were measured for each fungal sample; 98,369 raw tags were obtained through quality control, with 64,082 effective tags and a 62.51% effective rate of quality control. The sequences were clustered into OTUs with 97% identity. A total of 5,210 bacterial OTUs and 1,813 fungal OTUs were obtained. In the annotation results, 1,847 (35.45%) bacterial OTUs and 1,291 (71.21%) fungal OTUs were annotated to the genus level. We then calculated the α-diversity for bacteria and fungi as affected by the treatments (the CKs and Ms) and as related to environmental properties.

### Alpha diversity of the microbial community in the hyphosphere of *Morchella sextelata*

The rarefaction curves (Supplementary Figure 2) of all groups of bacteria and fungi approached a saturation plateau, indicating that the amount of data sequenced was sufficient to reflect the information for most microorganisms in the samples. The microbial richness and diversity between the groups were analyzed by Chao1 index and Shannon index (Supplementary Figure 2 and Supplementary Table 2.1). The significances were also analyzed by Wilcoxon’s test (Supplementary Tables 2.2, 2.3). After planting *Morchella*, the richness and diversity of both fungal and bacterial communities in the soil were significantly increased compared with the control groups (M1 vs. CK1 and M2 vs. CK2), indicating that the number of *Morchella*-related microorganisms increased over time.

### Bacterial community composition in hyphosphere soil with continuous cropping of *Morchella sextelata*

A total of 2,810 prokaryotic OTUs were shared by the five sample types (Supplementary Figure 3). Unique prokaryotic microorganisms were more abundant in the M samples than in the CK samples, and the unique prokaryotic microorganisms were more abundant in M1 than in CK1 and were more abundant in M2 than in CK2. For CK0, M1, and M2, M1 and M2 shared the highest number of OTUs (351), indicating that the composition of bacterial communities in M1 and M2 was highly similar. M1 and M2 shared 3,653 OTUs, and M1 had more unique OTUs (533) than M2 (505). For CK0, CK1, and CK2, there were 3,125 OTUs, and the number of unique OTUs was in the order CK0 (295) > CK1 (257) > CK2 (228), indicating that the number of unique OTUs in the CKs decreased year by year.

**FIGURE 2:**
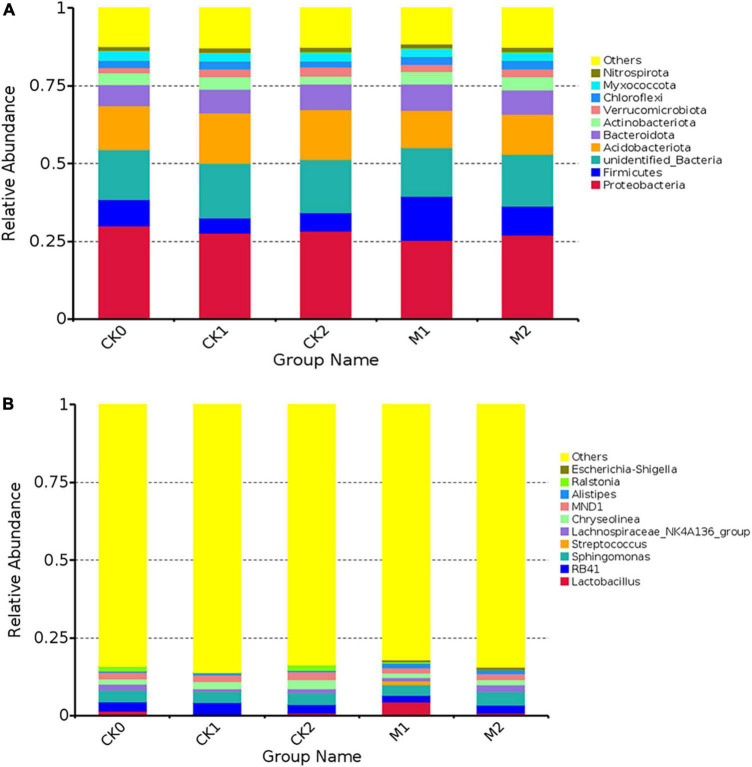
The composition of bacterial communities at the phylum level **(A)** and genus level **(B)** in the five types of samples. The 10 species with the highest abundance at each level were selected for each sample type. “Others” represents of the sum of the relative abundance of all other phyla (genus) other than these 10 phyla or genera.

To gain a deeper understanding of changes in bacterial community abundance and composition during continuous cropping of *M. sextelata*, bacterial communities were analyzed at the phylum level (Figure 2A) and the genus level (Figure 2B). We also assessed differences in the relative abundance of some bacteria in the five types of samples (Supplementary Table 3). At the phylum level, the dominant phylum of in each sample type was Proteobacteria. The abundance of Acidobacteriota was slightly lower in the Ms than in the CKs. The abundance of Firmicutes was 68.5% higher in M1 than in CK0, but decreased by 34.5% in M2; the trend in changes from CK0 to CK1 and CK2 was opposite to that from M1 to M2. There was no significant difference in the abundance of Proteobacteria among the five sample types, but at the order level, the abundance of Enterobacterales was much higher in the Ms than in the CKs, and was higher in M2 than in M1. In the Bacteroidota, the abundance of Bacteroidales was higher in the Ms than in the CKs, and the abundance of Cytohagales was higher in the CKs than in the Ms.

At the genus level, the dominant genus in M1 was *Lactobacillus*, while the dominant genus in the other four sample types was *Sphingomona*. The abundances of *Lactobacillus*, *Escherichia*, *Shigella*, and *Alitipies* were much higher in M1 than in CK0, but their abundances were similar among CK1, CK2, and CK3. Interestingly, the abundances of some bacterial genera had the opposite trend in M1 vs. M2; For example, *Lactobacillus* and *Streptococcus* increased in M1 and then decreased in M2, and *RB41* and *Streptococcus* decreased in M1 and then increased in M2, such that the abundances of these bacteria in M2 were similar to their abundance in CK0.

### Fungal community composition in the hyphosphere soil with continuous cropping of *Morchella sextelata*

There were 510 eukaryotic OTUs shared among the five sample types (Supplementary Figure 4). The unique eukaryotic microorganisms were more abundant in the Ms than in the CKs, and were more abundant in M1 than in CK1 and in M2 than in CK2. For CK0, M1, and M2, M1 and M2 shared the most OTUs (263), indicating that the similarity in the composition of fungal communities was high for M1 and M2. M1 and M2 shared 936 OTUs, and the number of OTUs was higher in M2 (373) than in M1 (256). A total of 3,125 OTUs were shared by the three CKs, and the order of the number of OTUs was CK2 (161) > CK0 (149) > CK1 (126).

**FIGURE 3:**
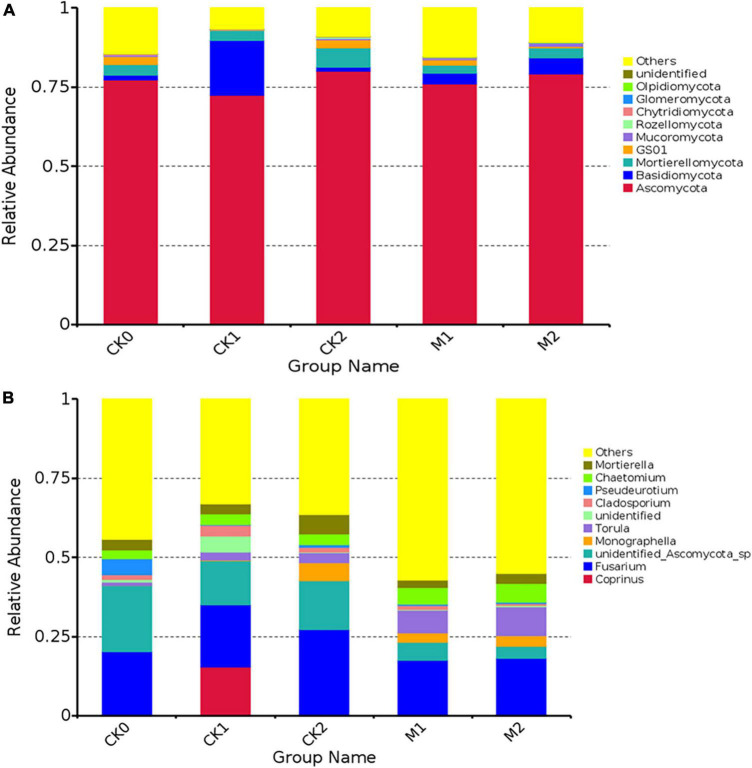
The composition of the fungal community at phylum level **(A)** and genus level **(B)** in the five types of samples. The 10 species with the highest abundance at the phylum or genus level were selected for each sample type. “Others” represents the sum of the relative abundances of all other phyla (genera) other than these 10 phyla or genera.

The fungal community was analyzed at the phylum level (Figure 3A) and genus level (Figure 3B). The dominant phylum for all five sample types was Ascomycota. We also assessed the differences in the relative abundance of some fungi in the sample types (Supplementary Table 3). The abundance of Basidiomycota was 140% higher in M1 than in CK0, and its abundance was nearly 11 times higher in CK1 than in CK0. The abundance of Mucoromycota was 85% higher in M2 than in M1, and the abundance of Glomeromycota was 168% higher in M1 than in CK0 and was 23% higher in M2 than in M1. In contrast to the changes in the abundance of these two phyla in the Ms, the changes in the CKs were small.

At the genus level, the dominant genus in CK0 was an *unidentified Ascomycota sp.*, and the dominant genus in the other sample types was *Fusarium*. The abundance of *Fusarium* was similar in CK1 and CK2, and was slightly lower in the Ms than in the CKs. The abundance of *Penicillium* was 157% higher in M1 than in CK0, and was 105% higher in M2 than in M1, but the variation in the abundance of *Penicillium* in the CKs was small. The abundance of *Trichoderma* was 422% higher in M1 than in CK0, but the variation of the abundance of *Trichoderma* in the CKs was small. In addition, the abundance of some common pathogenic fungi, such as *Fusarium oxysporum*, *Botrytis cinerea*, *Clonostachys rosea*, and *Aspergillus niger*, increased during continuous cropping of *M. sextelata*.

### Effects of continuous cropping on the microbial community structure in the *Morchella sextelata* hyphosphere

**FIGURE 4:**
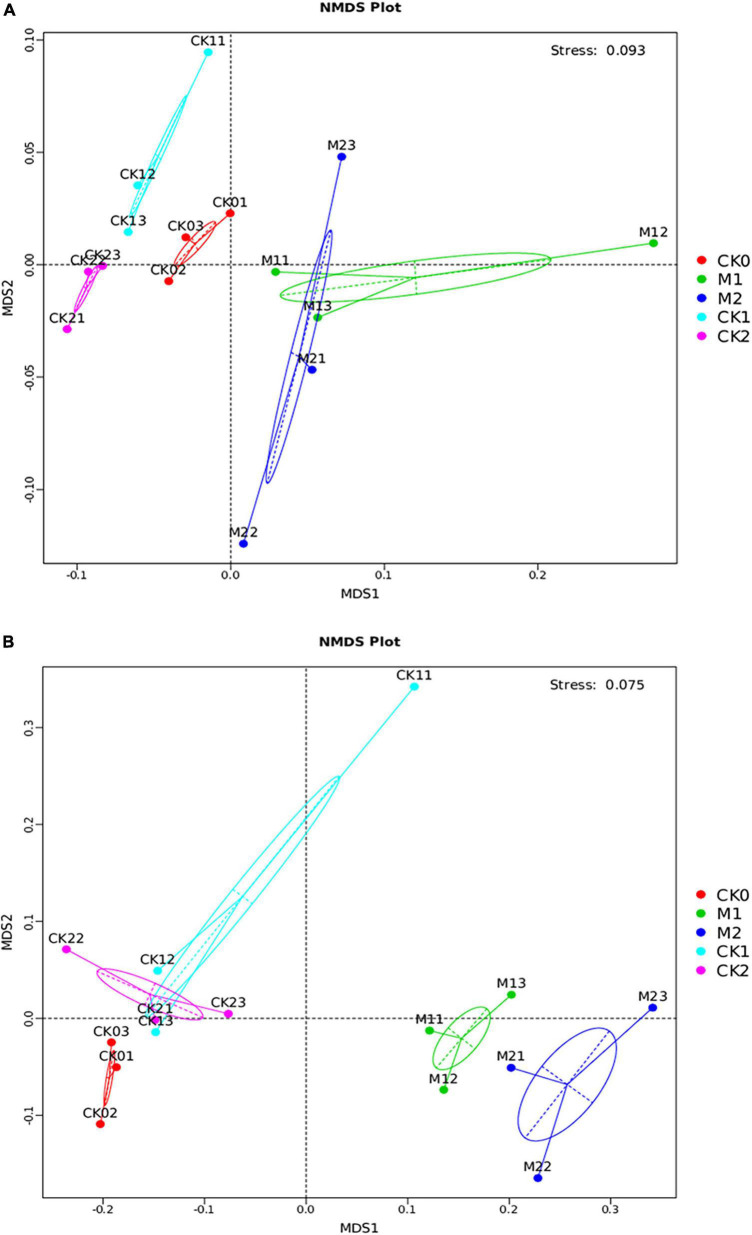
Differences in microbial community structure among the five sample types. Bray curtis NMDS analysis of bacterial community **(A)** and fungal community **(B)** at the OTU level. The top right corner of **(A,B)** shows the stress value of the NMDS analysis.

NMDS analysis and UMGPA clustering tree analysis were performed on the microbial communities in the five sample types. For both the bacterial community (Figure 4A) and the fungal community (Figure 4B), M1 and M2 were well separated from the CKs in the NMDS analysis, indicating that microbial community structure differed in soil in which *M. sextelata* had or had not been continuously cropped. The small distance between M1 and M2 indicated that the microbial community structures of M1 and M2 were similar. Similar results were obtained in the gate level analysis of the UMGPA clustering tree (Supplementary Figure 5). In these analyses, the microbial community structure was similar for the CKs, i.e., CK0, CK1, and CK2.

### Relationships between environmental factors and on bacterial and fungal community composition

**FIGURE 5:**
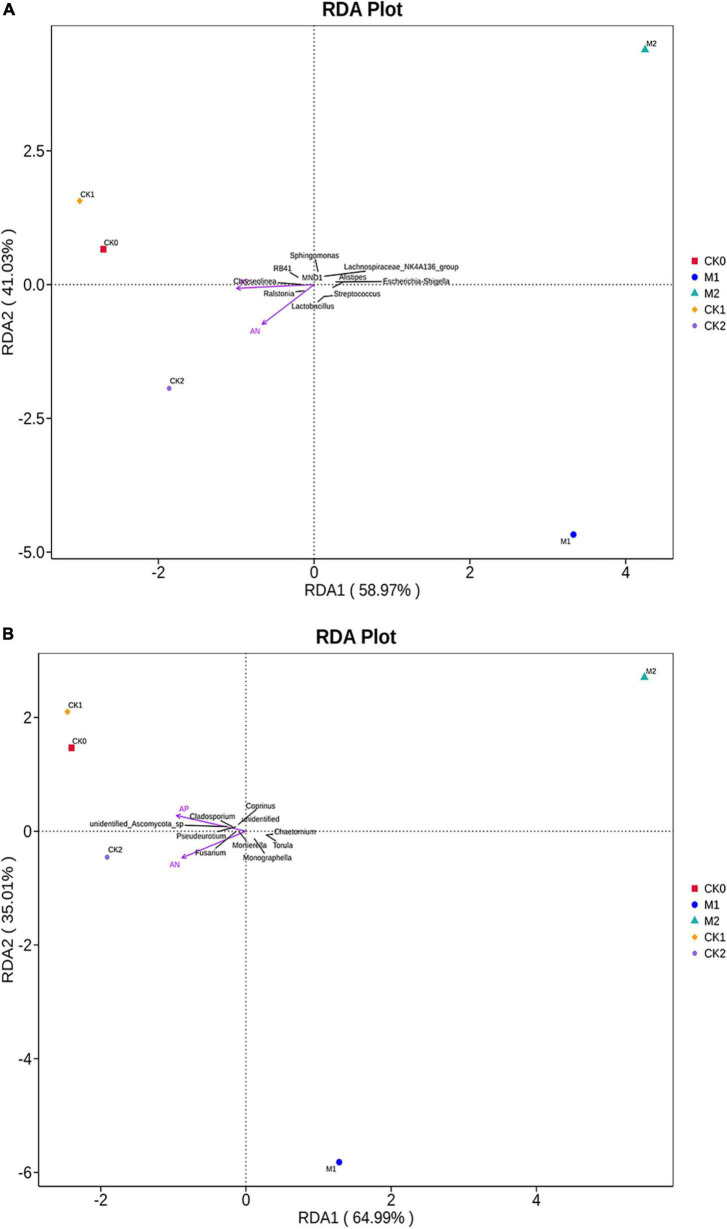
Redundancy analysis plots of the correlation between environmental factors (after Vif screening) and the **(A)** bacterial community and **(B)** fungal community in the five types of samples.

RDA analysis was performed on the bacterial and fungal communities (Figure 5). RDA components (RDA1 and RDA2) explained 59 and 41% of total variation in the bacterial community, and 65 and 35% of total variation in the fungal community. In addition, *r*^2^ and *P*-values were calculated to investigate the possible relationships between soil environmental factors and microbial community composition. Among soil environmental factors, the bacterial community composition (Figure 5A) was significantly related to AN (*r*^2^ = 0.984, *P* = 0.025) and AP (*r*^2^ = 0.973, *P* = 0.033), and the fungal community composition (Figure 5B) was also significantly related to AN (*r*^2^ = 0.995, *P* = 0.05) and AP (*r*^2^ = 0.985, *P* = 0.033) in the five types of samples.

Spearman rank correlation analysis was used to determine the relationships between environmental factors and the α-diversity indices of the key microorganisms (Supplementary Table 4). In the bacterial community, *Bacillus* was negatively correlated with AN (*P* < 0.05), TP (*P* < 0.01), and OM (*P* < 0.05), and *Lactobacillus* was negatively correlated with TP (*P* < 0.01). The α-diversity index of the bacterial community was negatively correlated with AN, TP, TN, and OM. The abundances of most pathogenic fungi were negatively correlated with environmental factors; more specifically, the abundances of *Penicillium*, *Stachybotrys*, and *Trichoderma* and the fungal community α-diversity index were negatively correlated with most environmental factors.

### Effects of continuous cropping of *Morchella sextelata* on the predicted functions of soil microorganisms in the hyphosphere

**FIGURE 6:**
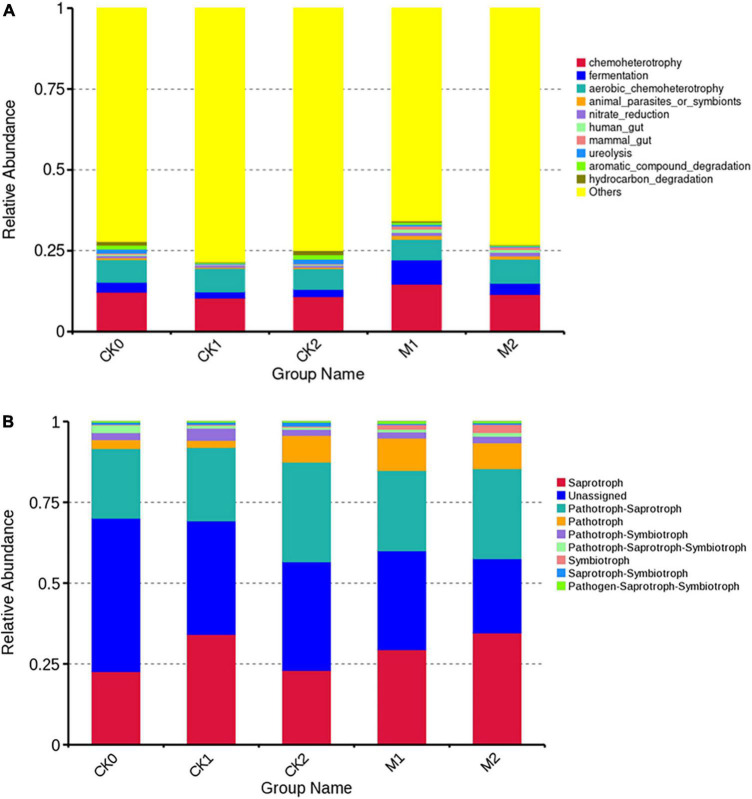
Ecological function relative abundance of bacterial community **(A)** and fungal community **(B)**.

**FIGURE 7:**
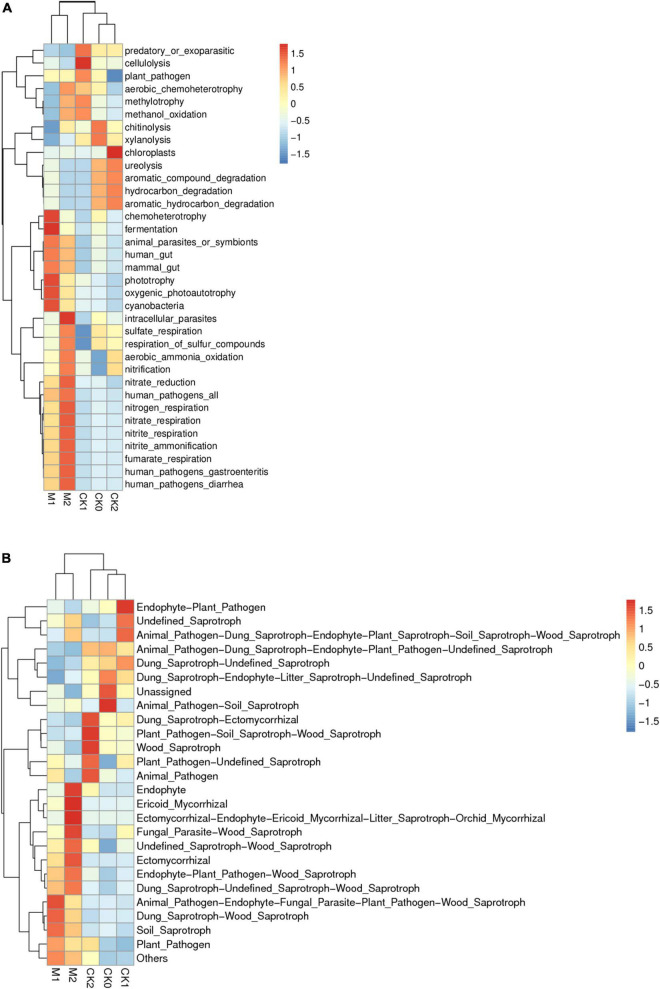
Ecological function heatmap of bacterial community **(A)**; ecological function heatmap of fungal community **(B)**.

The results of PICRUSt showed the KEGG metabolic pathway Level2 contains seven classes and 41 metabolic pathways (Supplementary Figure 6). The pathway amino acid metabolism was the most enriched, followed by the pathways’ membrane transport, carbohydrate metabolism, replication and repair, energy metabolism, and undesirable characteristics. FAPROTAX was then used to predict the ecological functions of bacteria. The functions chemoheterotrophy and fermentation were more enriched in M1 than in the other four sample types (Figure 6A). The heatmap (Figure 7A) indicates that genes related to N cycle functions were significantly more abundant in the Ms than in the CKs, and were more abundant in M2 than in M1. Genes related to sulfate consumption and intracellular parasites also increased year by year during the continuous cropping of *M. sextelata*. Genes associated with chemoheterotrophy, fermentation, phototrophy, oxygenic photoautotrophy, and cyanobacteria were more abundant in the Ms than in the CKs, but were less enriched in M2 than in M1. Genes associated with cellulolysis, chitunolysis, xylanolysis, chloroplasts, and ureolysis were more abundant in the CKs than in the Ms. Principal components analysis (Supplementary Figure 7) showed that some bacterial community functions differed between the CKs and the Ms.

The results of FunGuild (Figure 6B) showed that the relative abundances of saprotrophs and pathograph-saprotrophs were high in all groups except unassigned. Pathotrophs were more abundant in the Ms than in the CKs, and symbiotrophs were more abundant in M2 than in M1. According to the heatmap (Figure 7B), wood saprotrophs were more abundant in the Ms than in the CKs, and ectomycorrhizal fungi, endophytic fungi, and parasitic fungi were more abundant in M2 than in M1 or the CKs. Soil saprotrophs and plant pathogens were more abundant in M1 than in M2. PCA was performed based on the statistical results of the abundance of functional annotations in the database (Supplementary Figure 7). The close distances in the dimension reduction diagram indicate that the functions of fungi were similar in M1 and M2. Similarly, the close distances in the dimension reduction diagram indicate that the fungi in CK0, CK1, and CK2 have similar functions. The long distances between the Ms and the CKs indicate that the fungi have different functions in the Ms than in the CKs.

## Discussion

### Analysis of soil allelochemicals and physicochemical properties

In a Petri dish assay, we found that the growth and development of *M. sextelata* mycelium did not differ among the five sample types. However, more *M. sextelata* sclerotia formed near the M2 sample type than near the other four sample types. A possible reason is that exudates in the *M. sextelata* hyphosphere promoted the growth and development of *M. sextelata* sclerotia. The release of such allelochemicals could lead to an excessive loss of available nutrients, resulting in reduced production when *M. sextelata* is continuously cropped. Future studies will identify the allelochemicals and explore their properties and driving mechanisms through metabolome and transcriptome analysis.

In the current study, the continuous cropping of *M. sextelata* resulted in a decrease in available nutrients and in soil pH. A previous study found that changes in soil physicochemical properties in the *M. sextelata* hyphosphere are closely related to changes in the microbial community and especially the bacterial community (Zhang et al., 2019). We found that AN was significantly lower in M2 than in M1 or CK0. Soil N content can affect the growth of *Morchella* spp. and especially the growth of *Morchella* spp. sclerotia (Volk and Leonard, 1989; Mu, 2019). We infer that the allelochemicals in M2 soil enhanced the growth of *M. sextelata* sclerotia, resulting in the consumption of a large amount of AN, such that the content of AN was significantly reduced in M2.

### Analysis of the microbial community composition in the hyphosphere of continuously cropped *Morchella sextelata*

For the bacterial community, the abundance of *Bacillus* was 151% higher in M1 than in CK0, but was 70% lower than in M2. *Bacillius* spp. substantially affected the growth and development of *Morchella rufobrunnea* fruiting bodies in a recent study (Longley et al., 2019). This is because *Bacillus* can not only inhibit the pathogenic fungus *Trichoderma harzianum* in *Pleurotus* (Velázquez-Cedeño et al., 2008), but can also produce a lipopeptide that inhibits pathogenic microorganisms such as *Fusarium oxysporum* (Sarwar et al., 2018). The results of the current study suggest that although *Bacillus* spp. may have increased the growth of *M. sextelata* during the first year of cropping, the abundance of *Bacillus* spp. declined in the second year, which may have increased the risk that *M. sextelata* was attacked by other microorganisms. The abundance of *Bacillius* may have decreased in M2 because of competition with *Clostridium* or the influence of environmental factors. The abundance of *Lactobacillus* was 192% higher in M1 than in CK0, but was 79% lower in M2 than in M1. Polysaccharides produced by edible fungi stimulate *Lactobacillus* growth and provide fermentation substrates for *Lactobacillus* (Nowak et al., 2018; Tohno et al., 2019). In the current study, *M. sextelata* grew normally in the first year and may have produced a large amount of polysaccharides, which may have increased the abundance of *Lactobacillus*. At the same time, *Lactobacillus* can produce lactic acid, which could reduce the pH of the soil. The decrease of soil pH would provide favorable conditions for the growth of pathogenic fungi such as *Trichoderma*, *Mucor*, and *Penicillium*, thereby increasing the probability that *M. sextelata* was infected by pathogens. In the second year of cropping, however, *M. sextelata* growth was decreased, which may have led to a sharp decrease in the content of polysaccharides in the soil and therefore in the abundance of *Lactobacillus*.

In the current study, we found that changes in the abundance of pathogenic fungi with the continuous cultivation of *M. sextelata* had three patterns. In one pattern, the cultivation of *M. sextelata* in the first year resulted in an increase in the abundance of pathogenic fungi, i.e., the abundance of pathogenic fungi was higher in M1 than in CK0. It is therefore possible that this increase in pathogenic fungi led increased infection of *M. sextelata* by these fungi in the second year of cropping. The pathogenic fungi that increased in abundance included *Penicillium*, *Trichoderma*, *Aspergillus sp.*, *Fusarium oxysporum*, *Botrytis cinerea*, and *Clonostachys rosea*. *Penicillium* is a common pathogenic fungus in the production of edible fungi. In this study, the abundance of *Penicillium* increased with the years of continuous cropping of *M. sextelata*. *Penicillium* was previously found to interfere with the cultivation of *Pleutus ostreatus*, *Lentinus edodes*, and other mushrooms (Xiang, 2007; Liu, 2017). By inhibiting the mycelial growth of edible fungi, *Penicillium* can prevent the fungi from forming fruiting bodies or might cause the fruiting bodies to rot and become brown. *Trichoderma* is also a main pathogen of *Morchella* (Liu, 2017; Chen et al., 2018) found that the abundance of *Trichoderma* was higher in a soil in which *Morchella* stipe rot disease occurred than in a soil without the disease. In our study, *Trichoderma* abundance was much higher in the Ms than in the CKs. *Aspergillus* sp. can cause white mildew of *Morchella* (Yu et al., 2020). In our study, *Aspergillus* abundance was 105% higher in M1 than in CK0, and was 62% higher in M2 than in M1. In addition to causing disease of plant roots, *Fusarium* spp. can also cause disease of *Morchella* (Scherm et al., 2013; SMa et al., 2013; Guo et al., 2016). For example, *Fusarium nematophilum* was identified as the pathogen of stipe rot disease of *M. sextelata* (Liu et al., 2021b). Although this species was not identified in the current study, other species might have a similar effect. We found that the abundance of *F. oxysporum* increased year by year with *M. sextelata* cultivation, i.e., its abundance was 92% higher in M1 than in CK0 and 19% higher in M2 than in M1. The *F. oxysporum* species complex had been reported to infect more than 120 plant species (Pietro et al., 2003; Michielse and Rep, 2009; Li et al., 2021b). *Botrytis cinerea* has a wide host range and can cause disease in more than 200 plant species (Williamson et al., 2007; Cheung et al., 2020). The abundance of *B. cinerea* was 66% higher in M1 than in CK0, and was 40% higher in M2 than in M1; in contrast, there was little change in *B. cinerea* abundance in the CKs. *Clonostachys rosea* is considered a pathogen of *Cordyceps militaris* and naked barley (Li et al., 2018; Liu et al., 2021a); in the current study, its abundance was 36% greater in M1 than in CK0 and was 42% greater in M2 than in CK0. The increase in the abundance of these pathogenic fungi began in the first year, which increased the chances of infection in the next year.

A second pattern of increased abundance of pathogenic fungi was also detected in the current study. In this case, the abundance of pathogenic fungi was similar in M1 and CK0 but was greater in M2 than in M1 or CK0; in other words, these fungi did not increase until the second year of *M. sextelata* cultivation. Pathogenic fungi that conformed to this pattern included *Mucor, Stachybotrys, Aspergillus niger*, and others. *Mucor* is a common pathogen of edible genus such as *Agrocybe aegerita* and *Morchella* (Choi et al., 2010; Liu, 2017). *Mucor* is a common fungus that infects *via* spores. In our study, the abundance of *Mucor* was 1,158% higher in M2 than in M1 but did not differ greatly between M1 and the CKs. *Stachybotrys* usually has a high abundance in the soil where *Morchella* stipe rot disease occurs (Chen et al., 2018). In our study, the abundance of *Stachybotrys* was 189% higher in M2 than in M1, while there was little difference in its abundance between CK0 and M1 or between CK1 and CK2. *Aspergillus nige*r is also a common pathogen of edible fungi including *Morchella* and *Cordyceps militaris* (Liu, 2017; Liu et al., 2021a). In our study, the abundance of *A. nige*r was 200% higher in M2 than in M1.

In the third pattern, the abundance of pathogenic fungi increased over time whether *M. sextelata* was present or not. The fungus *Cephalotrichum* exhibited this pattern and is a potential pathogen of *Agaricus bisporus* (Fergus, 1978; Dugan et al., 2012). *Cephalotrichum* may parasitize *Morchella* mycelium or may inhibit the development of its fruiting bodies (Tan et al., 2021). *Cephalotrichum* abundance did not significantly differ between CK0 and M1, but it was 78% higher in M2 than in M1. However, its abundance in CK2 also increased by 69% compared with CK1, indicating that *Cephalotrichum* was also apparently increasing in response to environmental factors that were common to the CKs and the Ms. Therefore, the spores of pathogenic fungi such as *Cephalotrichum* may spread in the environment, it will increase the risk of infection by *Morchella*.

The abundance of cellulose-degrading microorganisms was also interesting. The abundance of cellulose-degrading bacteria such as *Cellvibrio* and *Cytohaga* was higher in the CKs than in the Ms. At the same time, the abundance of cellulose-degrading fungi such as *Chaetomium* and *Trichoderma* was higher in the Ms than in the CKs. Cellulose-degrading bacteria and fungi in soil may have a competitive relationship in different soil (Eichorst and Kuske, 2012). Perhaps the cultivation of *M. sextelata* altered the soil environment, increasing the competition between cellulose-degrading fungi and bacteria. Bacteria were more dominant than fungi in the CKs, while fungi were more dominant than bacteria in the Ms.

### Effects of environmental factors on microbial community structure

RDA analysis in the current study indicated that AN and AP contents in soil were strongly related to the structure of the bacterial community and fungal communities when *M. sextelata* was continuously cropped. According to Spearman rank correlation analysis, most of the measured environmental factors were significantly related to the diversity and richness of microbial communities. The relationships between environmental factors and pathogenic fungi were mostly negative correlated; the abundances of *Penicillium*, *Stachybotrys*, and *Trichoderma*, for example, were negatively correlated with multiple environmental factors. We suspect that the continuous cropping of *M. sextelata* decreases the content of AK, AP, AN in soil and reduces soil pH, resulting in an increase in the abundance of fungi that can infect *M. sextelata.*

### Functional predictive analysis of microbial community

Our KEGG analysis indicated that with continuous cropping of *M. sextelata* in all five sample types soil bacteria were enriched in the functions of amino acid metabolism, membrane transport, and carbohydrate metabolism. For soil bacteria, N cycle functions (e.g., nitrification and denitrification) were enriched significantly more in the Ms than in the CKs, and the enrichment of N cycle functions and sulfate respiration was higher in M2 than in M1. This may be because, as the richness and diversity of the bacterial community increases, so does the total amount of N that the bacterial community requires. Intracellular parasites increased year by year during the continuous cropping of *M. sextelata*, and M2 had the highest intracellular abundance, which would also increase the probability of *M. sextelata* infection. The prediction results of soil fungal functions prediction showed that enrichment of pathotrophs was higher in the Ms than in the CKs, and that enrichment of symbiotrophs was higher in M2 than M1. The enrichment of wood saprotrophs was greater in the Ms than in the CKs, and the enrichment of ectomycorrhizal, endophyte, and fungal parasites was greater in M2 than in M1 and the CKs. These increases are likely to increase the chances that *M. sextelata* will be negatively affected by competitors or pathogens with continuous cropping. PCA analysis showed that the functional structure of microbial communities and especially of fungal communities differed between the CKs and the Ms. This suggested that changes in the fungal community may help explain why *M. sextelata* production decreases with continuous cropping.

## Conclusion

Our results suggest that the decline in *M. sextelata* production with continuous cropping might result from several factors. Although the yield of *Morchella* fruiting bodies is generally high in the first year of cultivation, during that year the content of soil available nutrients decreases as does soil pH. The decrease in soil pH is associated with a gradual accumulation of pathogenic fungi such as *Penicillium*, *Trichoderma*, and *Aspergillus* in the second year of cultivation. Also in the second year of cultivation, the reduced fruiting rate of *Morchella* could lead to a decline in the abundance of *Bacillus* and other beneficial microorganisms in the soil. The increased abundance of pathogenic fungi and reduced abundance of beneficial microorganisms may explain why *M. sextelata* production decreases with continuous cultivation. Additional research is needed to determine whether these correlations are causal, i.e., whether increases in pathogenic fungi and decreases in beneficial microorganisms truly cause the decreases in *M. sextelata* production with continuous cultivation. The effects of allelochemicals on sclerotia production by *M. sextelata* also warrants further study.

## Data availability statement

The datasets presented in this study can be found in online repositories. The name of the repository and accession number can be found below: National Center for Biotechnology Information (NCBI) BioProject, https://www.ncbi.nlm.nih.gov/bioproject/, PRJNA822526.

## Author contributions

YX-D and GB: conceptualization. LY, GB, and BX: data curation. YX-D: formal analysis and funding acquisition. LY and GB: methodology and writing—original draft. GB and QJ: resources. LY: picture processing. YX-D and SA: writing—review and editing. All authors contributed to the article and approved the submitted version.

## Footnotes

1
http://qiime.org/scripts/assign_taxonomy.html

2
https://unite.ut.ee/
