# Supplementary material for: Determining why continuous cropping reduces the production of the morel Morchella sextelata
Source: Front Microbiol. 2022 Sep 12;13:903983. doi: 10.3389/fmicb.2022.903983 (PMC9510911; doi:10.3389/fmicb.2022.903983)
Supplement: Supplementary file 1 [file Data_Sheet_1.PDF]

Supplementary Material

1.1 Supplementary Figures

(A)

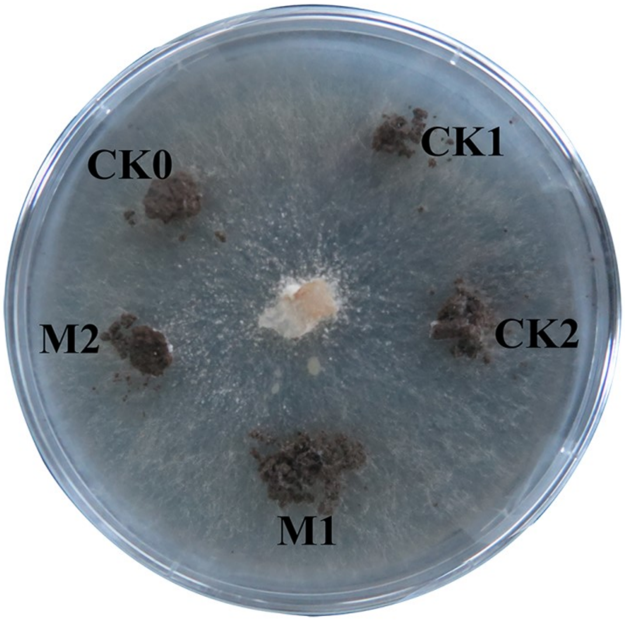

(B)

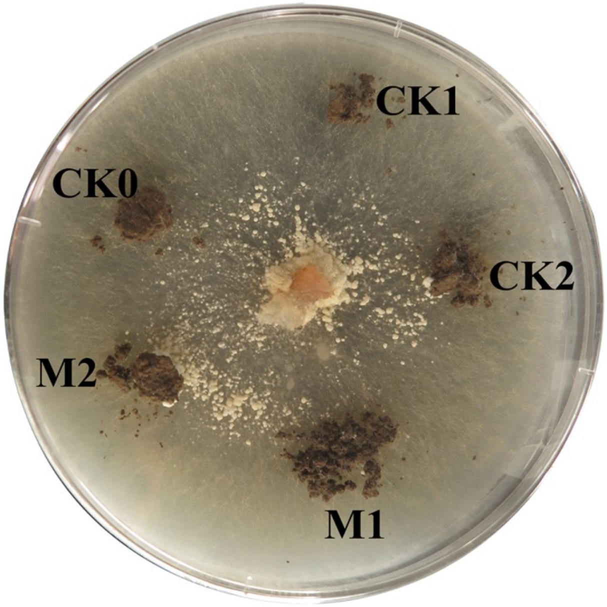

(C)

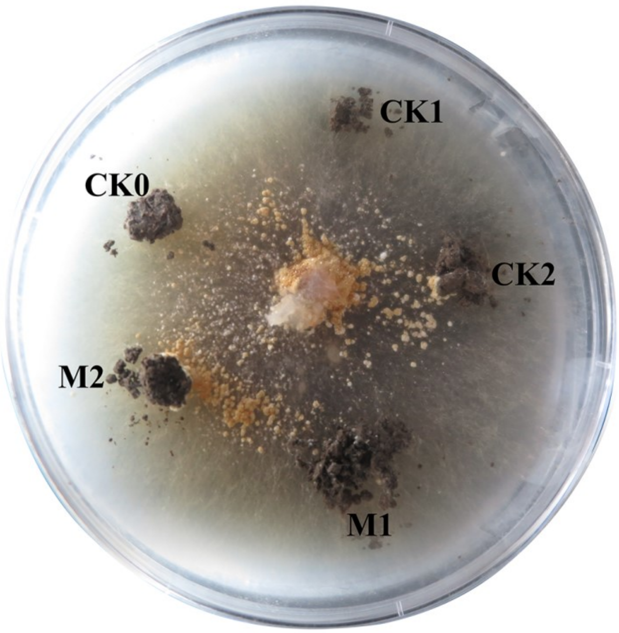

Supplementary Figure 1 | Photographs of *Morchella sextelata* on PDA plates with soil of the five sample types at **(A)** 4 day, **(B)** 6 day, and **(C)** 8 day after the fungus was added. The background of the tabletop on which the Petri plates sat was deleted to clarify the image.

(A)

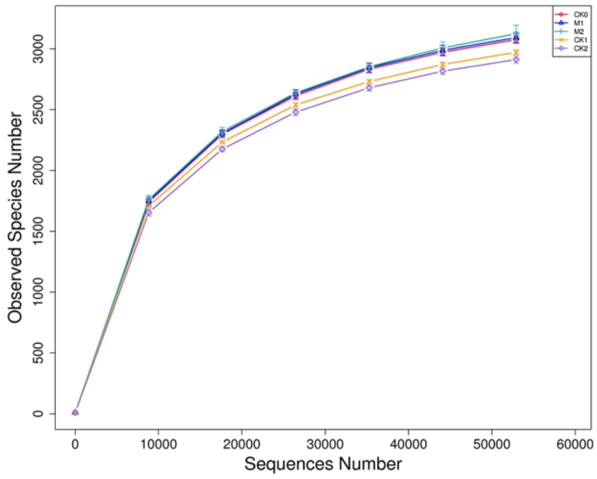

(B)

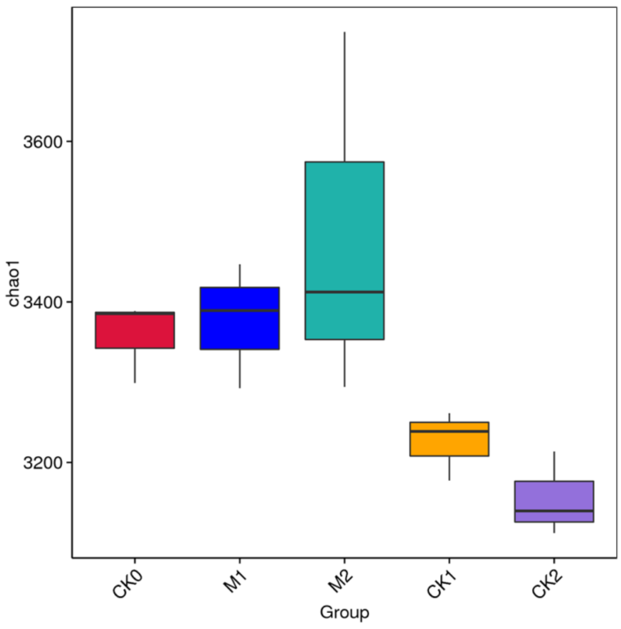
3600
3400
3200
chao1
CK0
M1
M2
CK1
CK2
Group

(C)

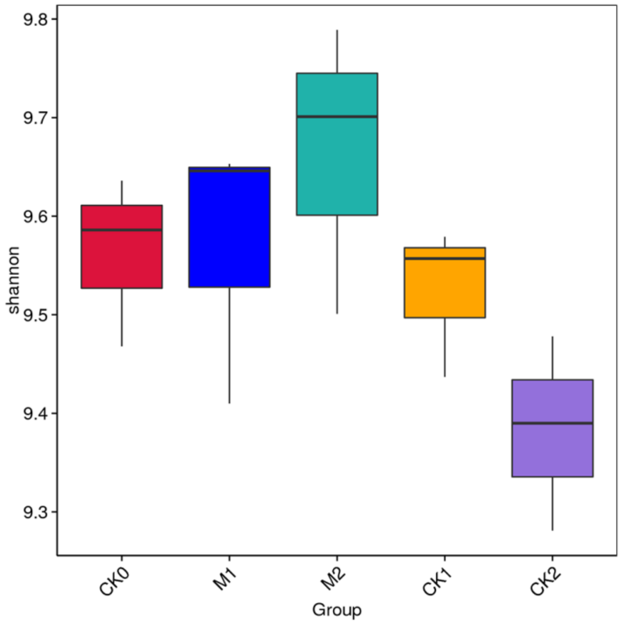
9.8
9.7
9.6
9.5
9.4
9.3
shannon
CK0
M1
M2
CK1
CK2
Group

(D)

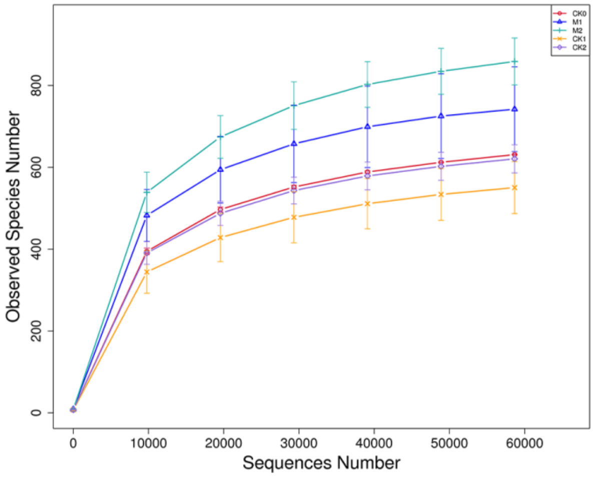
CK0
M1
M2
CK1
CK2
Observed Species Number
0
200
400
600
800
0
10000
20000
30000
40000
50000
60000
Sequences Number

(E)

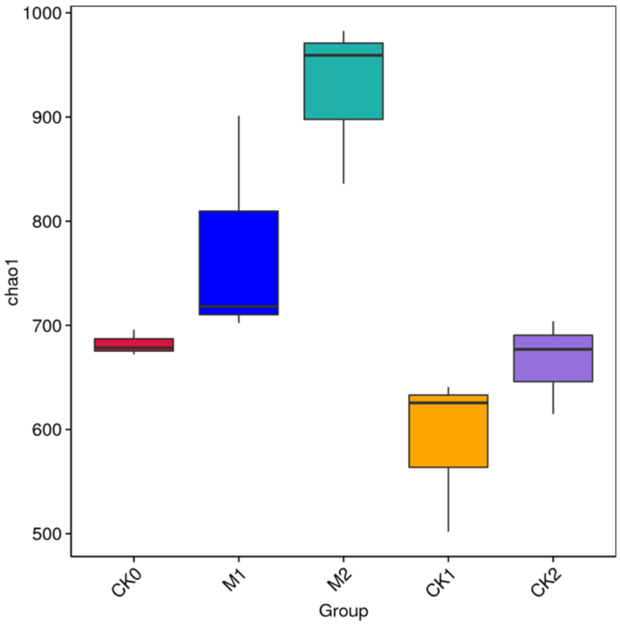
1000
900
800
700
600
500
chao1
CK0
M1
M2
CK1
CK2
Group

(F)

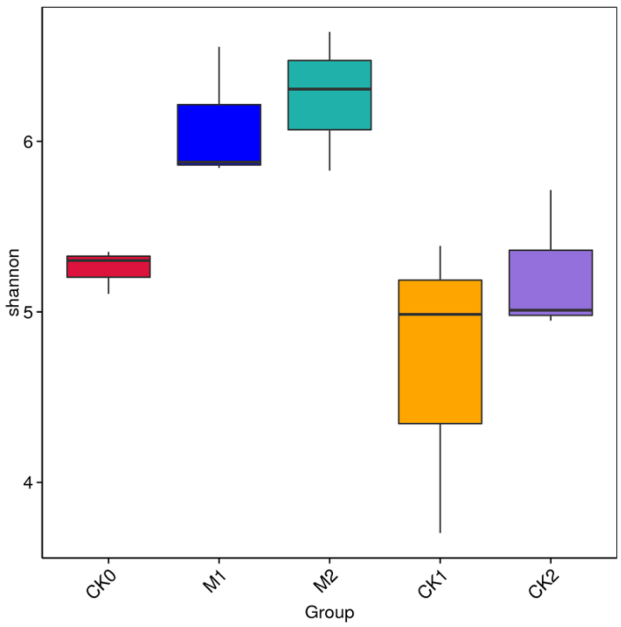

Supplementary Figure 2 | Bacterial community α-diversity in the five sample types. **(A)** Rarefaction curve; **(B)** Chao1; **(C)** Shannon. α-Diversity of fungal community. **(D)** Rarefaction curve; **(E)** Chao1 index; **(F)** Shannon index.

Supplementary Material

(A)

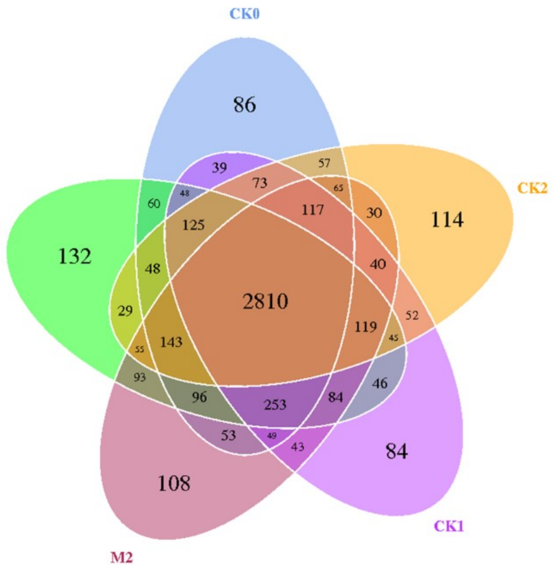

(B)

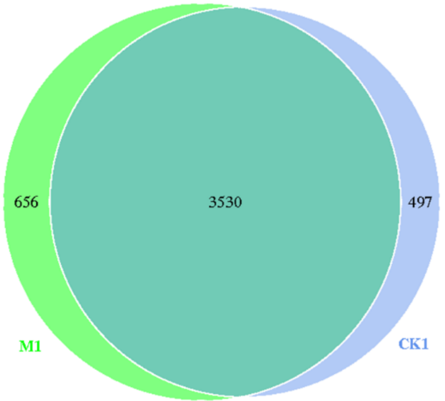

(C)

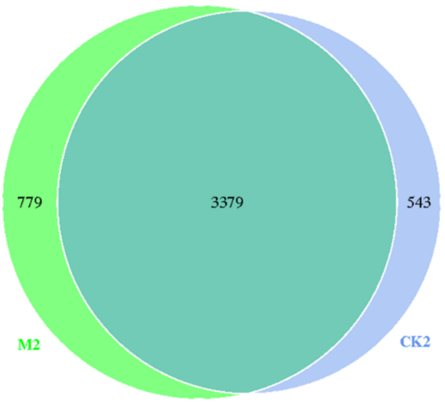

(D)

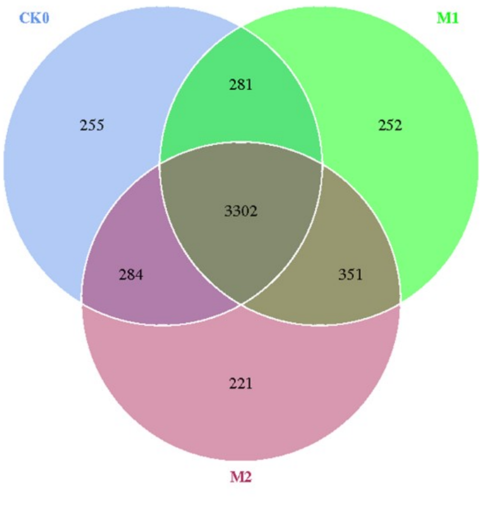

(E)

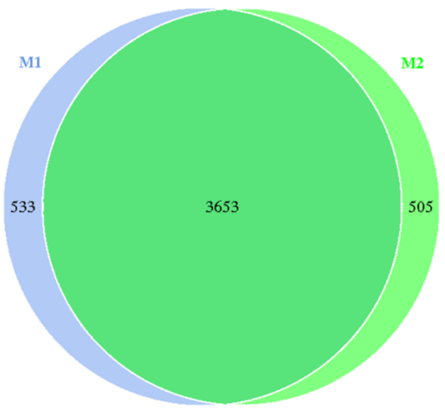

(F)

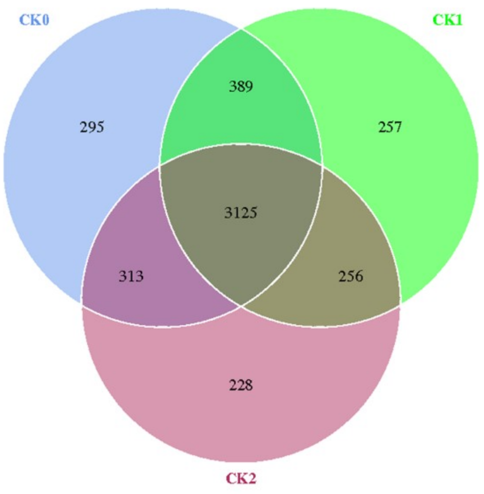

Supplementary Figure 3 | Venn diagram of bacterial OTU number between different groups. **(A)** CK0 - M1 - M2 - CK1 - CK2; **(B)** CK1 - M1; **(C)** CK2 - M2; **(D)** CK0 - M1 - M2; **(E)** M1 - M2; **(F)** CK0 - CK1- CK2.

(A)

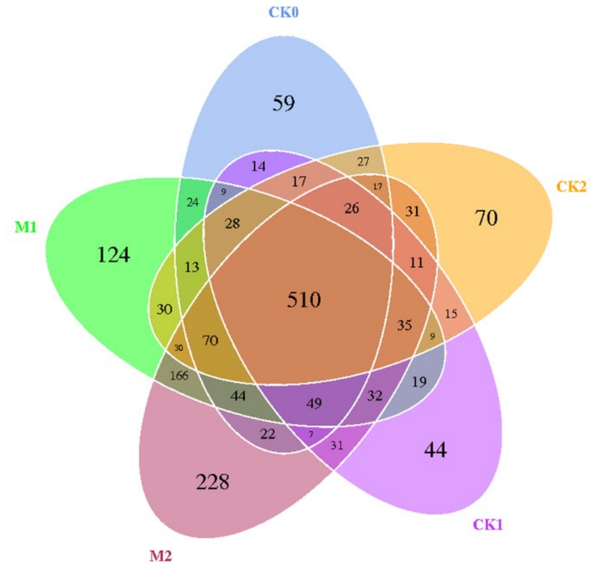

(B)

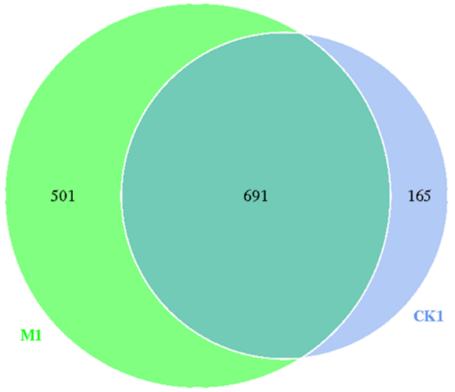

(C)

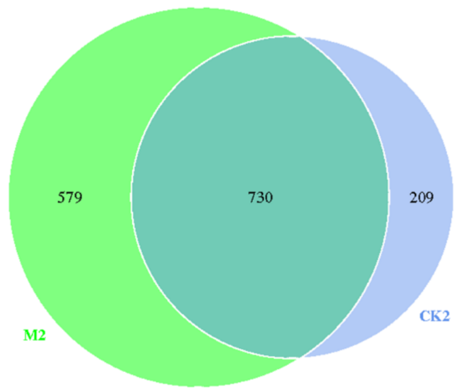

(D)

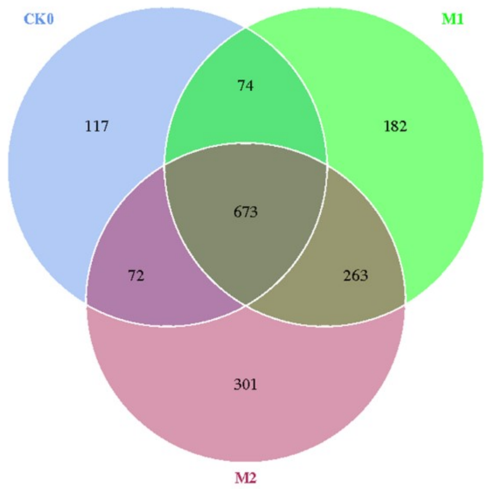

(E)

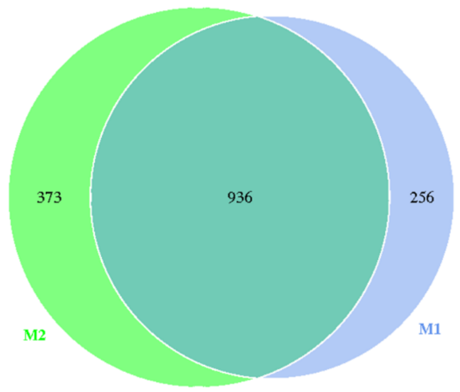

(F)

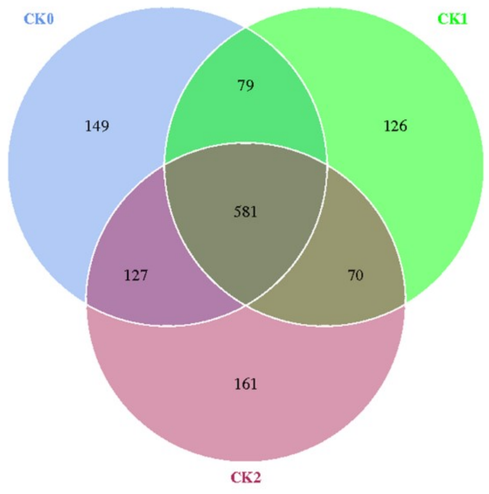

Supplementary Figure 4 | Venn diagram of fungal OTU number between different groups.**(A)** CK0 - M1 - M2 - CK1 - CK2; **(B)** CK1 - M1; **(C)** CK2 - M2; **(D)** CK0 - M1 - M2; **(E)** M1 - M2; **(F)** CK0 - CK1- CK2.

(A)

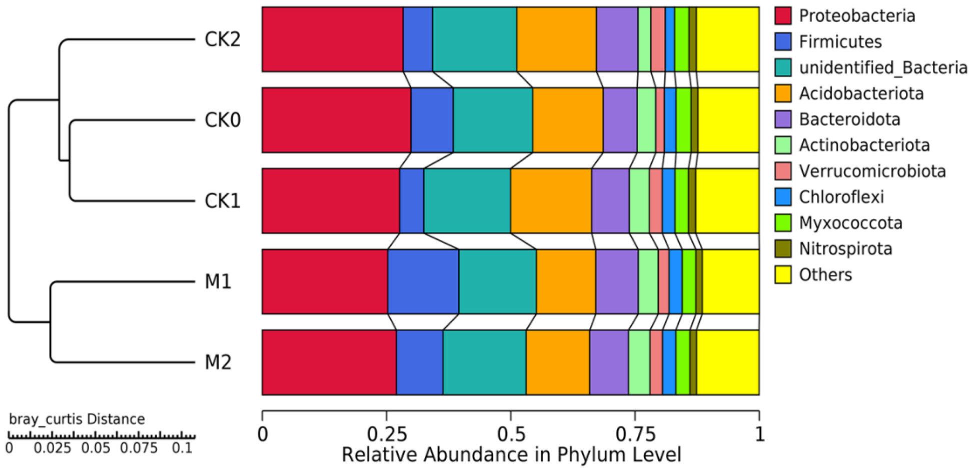

(B)

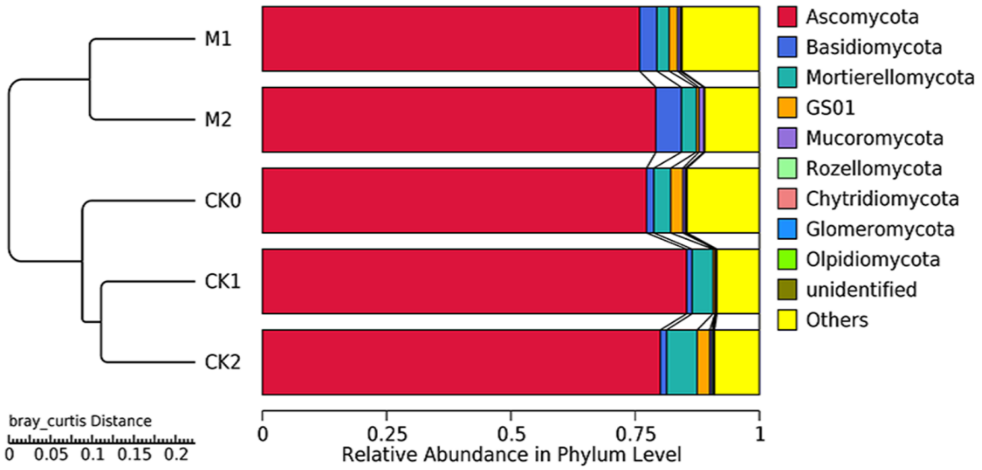

Supplementary Figure 5 | UPGMA clustering analysis of bacterial community **(A)** and fungal community **(B)** at the phylum level based on Bray–Curtis dissimilarity.

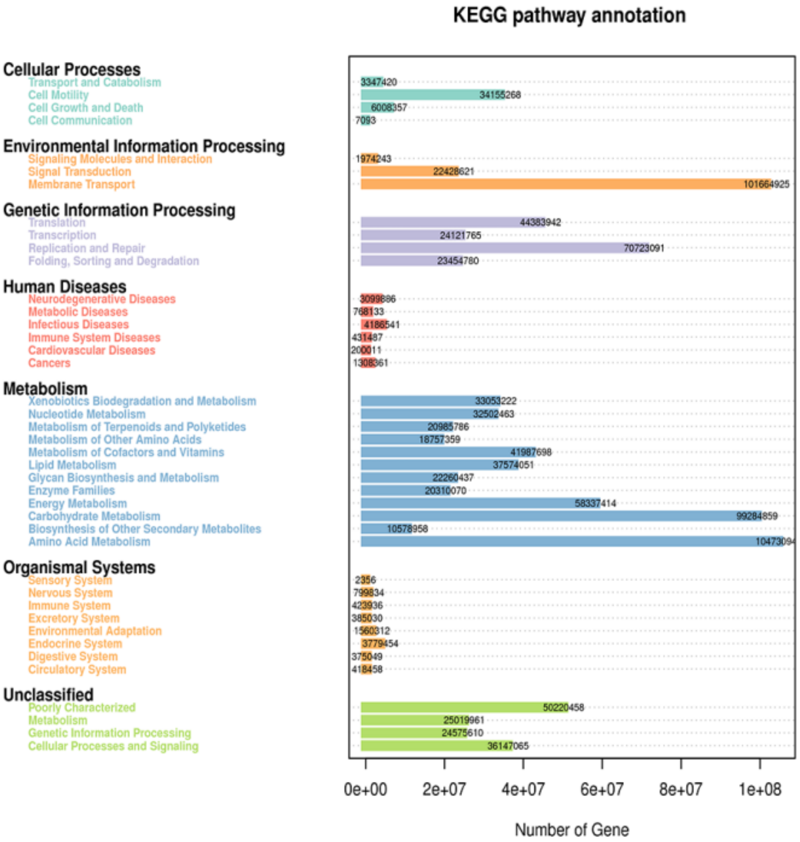

Supplementary Figure 6 | KEGG metabolic pathway level2 abundance of bacterial community.

(A)

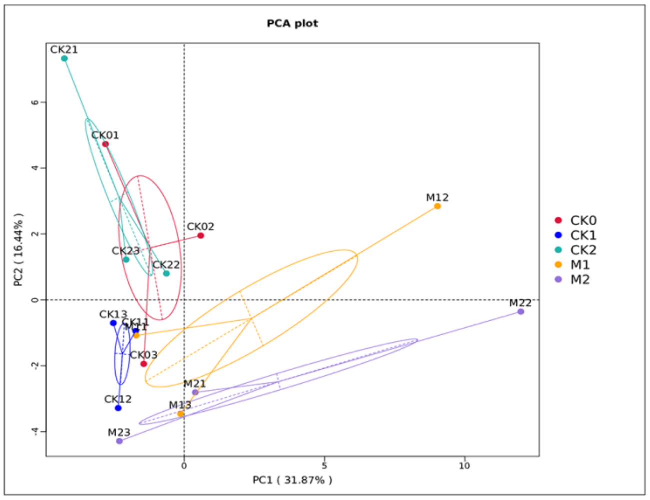

(B)

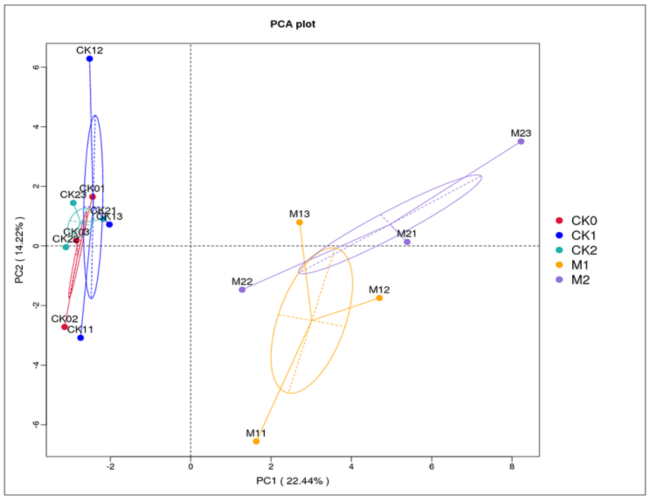

Supplementary Figure 7 | Principal components analysis of ecological function of bacterial community **(A)** and fungal community **(B)**.

1.2 Supplementary Tables

Supplementary Table 1.1 Yield and infection rate of *M. sextelata* in two years.

Boxes	Yield of M1(g)	fruiting body/diseased fruiting body	Infection rate	Yield of M2(g)	fruiting body/diseased fruiting body	Infection rate
1	221	9/1		111	5/4	
2	202	8/2		115	5/3	
3	243	10/0		132	6/2	
4	220	9/0		117	5/3	
5	219	9/1		112	5/2	
6	201	8/1		109	5/4	
7	231	10/1		112	5/3	
8	218	9/2		93	4/2	
9	240	10/1		113	5/3	
10	222	9/0		116	5/2	
Total	2217	91/9	9.0%	1130	50/28	35.9%

Supplementary Table 1.2 Paired Samples Test for the yield of *M. sextelata* in two years.

Paired Samples	Paired Differences							
	Mean	Std. Deviation	Std. Error Mean	Lower	Upper	t	df	Sig.(2-tailed)
M1 - M2	108.70000	13.07287	4.13401	99.34823	118.05177	26.294	9	.000

Lower and Upper are within the 95% confidence interval of the difference.

Supplementary Table 1.3 Paired Samples Test for the infection rate of *M. sextelata* in two years.

Paired Samples	Paired Differences							
	Mean	Std. Deviation	Std. Error Mean	Lower	Upper	t	df	Sig.(2-tailed)
M2 - M1	.26810	.07671	.02426	.21323	.32297	11.053	9	.000

Lower and Upper are within the 95% confidence interval of the difference.

Supplementary Table 2.1 Alpha Diversity Index of fungi and bacteria for all samples.

Sample type	**Bacterial alpha diversity index**		**Fungal alpha diversity index**	
	Chao1	Shannon	Chao1	Shannon
CK0	3357.663	9.563	682.178	5.253
CK1	3225.755	9.524	589.443	4.692
CK2	3155.021	9.383	665.352	5.224
M1	3376.2	9.57	773.919	6.092
M2	3480.958	9.664	926.043	6.259

Supplementary Table 2.2 Wilcoxon's test difference analysis of bacterial alpha diversity index.

Samples	Chao1			Shannon		
	Difference	*P*	sig.	Difference	*P*	sig.
CK1 - M1	-6.3333	0.0116	*	-2.3333	0.4737	
CK2 - M2	-9.6667	0.0008	***	-9.0000	0.0166	*

*Indicates P ≤ 0.05, **indicates P ≤ 0.01, ***indicates P ≤ 0.001.

Supplementary Table 2.3 Wilcoxon's test difference analysis of fungal alpha diversity index.

Samples	Chao1			Shannon		
	Difference	*P*	sig.	Difference	*P*	sig.
CK1 - M1	-8.3333	0.0014	**	-8.3333	0.0034	**
CK2 - M2	-7.6667	0.0024	**	-7.6667	0.0056	**

*Indicates P ≤ 0.05, **indicates P ≤ 0.01, ***indicates P ≤ 0.001.

Supplementary Table 3. Relative abundance of microorganisms mentioned in the article.

Microorganism	Sample type				
	CK0	M1	M2	CK1	CK2
**Bacteria**					
*p*_Acidobacteriota	0.141784	0.119502	0.127547	0.163007	0.160247
*p*_Firmicutes	0.084836	0.142987	0.093611	0.048538	0.058806
*p*_Proteobacteria	0.299234	0.252643	0.270004	0.276537	0.283737
*p*_Bacteroidota	0.068583	0.085611	0.078328	0.075859	0.083708
*o*_Bacteroidales	0.017009	0.038944	0.02848	0.018099	0.013695
*o*_Cytophagales	0.025614	0.019988	0.021097	0.030446	0.03832
*o*_Enterobacterales	0.020846	0.109688	0.108596	0.006055	0.015982
g_Bacillus	0.006621	0.009418	0.006205	0.004888	0.004933
g_Lactobacillus	0.015497	0.045243	0.009323	0.004044	0.008605
**Fungi**					
*p*_Ascomycota	0.772974	0.759249	0.791591	0.724293	0.800502
*p*_Glomeromycota	0.000159	0.000426	0.000523	0.000011	0.000108
*p*_Mucoromycota	0.004632	0.005155	0.009525	0.00083	0.004348
g_Cephalotrichum	0.007216	0.006815	0.010824	0.016637	0.01363
g_Fusarium	0.201657	0.172105	0.178715	0.197105	0.271786
g_Mucor	0.001248	0.000582	0.007321	0.000582	0.000416
g_Penicillium	0.00287	0.007365	0.015094	0.003421	0.003103
g_Stachybotrys	0.021281	0.023866	0.069014	0.047733	0.033214
g_Trichoderma	0.00545	0.028466	0.022948	0.004007	0.006558
s_Aspergillus niger	0.000499	0.000832	0.002496	0.000998	0.000666
s_Aspergillus sp	0.005657	0.012894	0.018884	0.004825	0.009067
s_Botrytis cinerea	0.00025	0.000416	0.000582	0.000083	0.001997
s_Clonostachys rosea	0.002658	0.003607	0.005125	0.002278	0.007024
s_Fusarium buharicum	0.000298	0.001691	0.001243	0.000746	0.001343
s_Fusarium oxysporum	0.009748	0.018701	0.022332	0.014971	0.019795
s_Fusarium sp	0.440515	0.38874	0.462449	0.62076	0.628618

Lowercase letters indicate the level of microorganisms. (p_: phylum; g_: genus; s_: species.)

Supplementary Table 4. Spearman correlation coefficients between environmental factors and key microorganisms (or α-diversity index).

Microorganism/α-diversity index	Physicochemical factors								
	AK	AP	AN	TK	TP	TN	OM	pH	HS
**Bacteria**									
*p*_Acidobacteriota	0.447	0.447	0.720**	0.480	0.660**	0.567*	0.720**	0.480	-0.120
*p*_Firmicutes	-0.338	-0.338	-0.655**	-0.393	-0.621*	-0.469	-0.655**	-0.393	0.218
*p*_Proteobacteria	0.698**	0.698**	0.349	0.611*	0.549*	0.567*	0.349	0.611*	-0.022
g_Bacillius	-0.273	-0.273	-0.546*	-0.306	-0.672**	-0.393	-0.546*	-0.306	0.491
g_Lactobacillus	-0.022	-0.022	-0.480	-0.153	-0.532*	-0.185	-0.480	-0.153	0.535*
g_Sphingomonas	-0.207	-0.207	-0.327	-0.218	-0.022	-0.262	-0.327	-0.218	-0.491
Chao1	-0.415	-0.415	-0.840**	-0.338	-0.739**	-0.666**	-0.840**	-0.338	0.185
Observed species	-0.469	-0.469	-0.873**	-0.371	-0.772**	-0.720**	-0.873**	-0.371	0.164
Shannon	-0.426	-0.426	-0.666**	-0.295	-0.565*	-0.611*	-0.666**	-0.295	0.011
**Fungi**									
*p*_Ascomycota	0.109	0.109	0.185	0.098	0.274	0.153	0.185	0.098	-0.240
g_Cephalotrichum	-0.077	-0.077	0.110	-0.066	0.129	0.011	0.110	-0.066	-0.219
g_Fusarium	0.360	0.360	0.720**	0.349	0.728**	0.546*	0.720**	0.349	-0.338
g_Mucor	0.017	0.017	-0.231	0.062	0.017	-0.130	-0.231	0.062	-0.248
g_Penicillium	-0.818**	-0.818**	-0.644**	-0.687**	-0.660**	-0.796**	-0.644**	-0.687**	-0.175

g_Stachybotrys	-0.683**	-0.683**	-0.551*	-0.650**	-0.468	-0.633*	-0.551*	-0.650**	-0.322
g_Trichoderma	-0.764**	-0.764**	-0.698**	-0.786**	-0.716**	-0.720**	-0.698**	-0.786**	-0.065
s_Aspergillus niger	-0.599*	-0.599*	-0.449	-0.449	-0.351	-0.599*	-0.449	-0.449	-0.365
s_Aspergillus sp	-0.498	-0.498	-0.306	-0.700**	-0.233	-0.301	-0.306	-0.700**	-0.350
s_Botrytis cinerea	-0.029	-0.029	0.405	-0.289	0.341	0.318	0.405	-0.289	-0.289
s_Clonostachys rosea	-0.166	-0.166	0.000	-0.354	0.199	0.011	0.000	-0.354	-0.554*
s_Fusarium buharicum	-0.230	-0.230	-0.005	-0.378	0.003	-0.044	-0.005	-0.378	-0.241
s_Fusarium oxysporum	-0.606*	-0.606*	-0.229	-0.775**	-0.182	-0.333	-0.229	-0.775**	-0.481
s_Fusarium sp	0.131	0.131	0.589*	0.131	0.588*	0.360	0.589*	0.131	-0.426
Chao1	-0.622*	-0.622*	-0.775**	-0.731**	-0.638*	-0.644**	-0.775**	-0.731**	-0.153
Observed species	-0.650**	-0.650**	-0.764**	-0.764**	-0.669**	-0.650**	-0.764**	-0.764**	-0.109
Shannon	-0.676**	-0.676**	-0.775**	-0.709**	-0.750**	-0.709**	-0.775**	-0.709**	0.011

*Indicates $P \leq 0.05$, **indicates $P \leq 0.01$.

Lowercase letters indicate the level of microorganisms. (p_: phylum; g_: genus; s_: species.)
